# Applicability of graded prognostic assessment of lung cancer using molecular markers to lung adenocarcinoma patients with brain metastases

**DOI:** 10.18632/oncotarget.19980

**Published:** 2017-08-07

**Authors:** Hongwei Li, Jianhong Lian, Songyan Han, Weili Wang, Haixia Jia, Jianzhong Cao, Xiaqin Zhang, Xin Song, Sufang Jia, Jiwei Ren, Weihua Yang, Yanfeng Xi, Shengmin Lan

**Affiliations:** ^1^ Department of Radiation Oncology, Shanxi Provincial Cancer Hospital, Shanxi Medical University, Taiyuan, Shanxi Province 030013, People’s Republic of China; ^2^ Department of Surgery, Shanxi Provincial Cancer Hospital, Shanxi Medical University, Taiyuan, Shanxi Province 030013, People’s Republic of China; ^3^ Department of Chemotherapy, Shanxi Provincial Cancer Hospital, Shanxi Medical University, Taiyuan, Shanxi Province 030013, People’s Republic of China; ^4^ Department of Pathology, Shanxi Provincial Cancer Hospital, Shanxi Medical University, Taiyuan, Shanxi Province 030013, People’s Republic of China; ^5^ Department of Medical Imageology, Shanxi Provincial Cancer Hospital, Shanxi Medical University, Taiyuan, Shanxi Province 030013, People’s Republic of China

**Keywords:** lung cancer, adenocarcinoma, brain metastass, gene alterations, prognosis

## Abstract

Several scoring systems are available to estimate prognosis and assist in selecting treatment methods for non-small cell lung cancer (NSCLC) patients with brain metastasis, including recursive partitioning analysis (RPA), basic score for brain metastases (BS-BM), and diagnosis-specific graded prognostic assessment (DS-GPA). Lung-molGPA is an update of the DS-GPA that incorporates EGFR and/or ALK mutation status. The present study tested the applicability of these four indexes in 361 lung adenocarcinoma patients with brain metastasis. Potential predictive factors in our independent multivariate analysis included patient age, Karnofsky performance status, EGFR and ALK mutation status, and use of targeted therapy. In the log-rank test, all four systems predicted overall survival (OS) (*P*<0.001). Harrell^’^s C indexes were 0.732, 0.724, 0.729, and 0.747 for RPA, BS-BM, DS-GPA, and Lung-molGPA, respectively. Our results confirmed that the Lung-molGPA index was useful for estimating OS in our patient cohort, and appeared to provide the most accurate predictions. However, the independent prognostic factors identified in our study were not entirely in agreement with the Lung-molGPA factors. In an era of targeted therapy, Lung-molGPA must be further updated to incorporate more specific prognostic factors based on additional patient data.

## INTRODUCTION

Brain metastases are among the leading causes of morbidity and mortality in patients with non-small cell lung cancer (NSCLC) [1]. Brain metastasis (BM) risk in NSCLC is 40% over the course of the disease [2, 3]. The prognosis for patients with BMs is generally poor; without treatment, median survival is estimated at 1–2 months. With whole brain radiotherapy (WBRT), the standard treatment for BMs regardless of histological subtype, median survival is 4–6 months [4, 5]. Several prognostic scoring systems based on independent prognostic factors have been developed to evaluate pretreatment variable contributions, help in selecting the appropriate treatment for individual patients, and guide future research. Widely accepted prognostic systems include, Recursive Partitioning Analysis (RPA), Basic Score for Brain Metastases (BS-BM), and the Graded Prognostic Assessment Index (GPA) [6-8]. However, prognostic factors vary with cancer type and histological subtype. Many cancers that commonly result in BMs, including NSCLC, small-cell lung cancer, breast cancer, melanoma, and renal carcinoma, involve different prognostic factors. Accordingly, Sperduto, *et al.* developed a diagnosis-specific prognostic factors index (DS-GPA) [9]. The NSCLC GPA includes age, Karnofsky performance status (KPS), extracranial metastases, and number of BMs. Although the DS-GPA stratified patients with BMs by primary site and highlighted characteristics of tumors that metastasize to the brain, the various primary tumor genotypes did not fit within the prognostic classification system. Additionally, gene alterations were also important prognostic factors for BMs [10-13].

Prognoses of lung adenocarcinoma patients with BMs are often associated with gene mutation status, including mutations in the epidermal growth factor receptor (EGFR), anaplastic lymphoma kinase-rearranged (ALK), and V-Ki-ras2 Kirsten rat sarcoma viral oncogene homolog (kRAS) [10, 14, 15]. To further refine the NSCLC-specific GPA, a new prognostic system, Lung-molGPA, was proposed based on 2,186 NSCLC patients with BMs. This new system included not only age, KPS, extracranial metastases, and number of BMs, but also EGFR and ALK mutation status [16, 17].

The present study reappraised the new Lung-molGPA index to determine its applicability in a Chinese patient cohort. The resultant scores were compared with those from three other published prognostic systems (RPA, BS-BM, DS-GPA), and predictive values were assessed.

## RESULTS

### Patient characteristics and treatment

By the end of the follow-up period for the 361 patients diagnosed with BMs from lung adenocarcinoma, 160 (44.3%) had confirmed EGFR mutations and 10 (2.8%) had ALK mutations; one patient had both. Pretreatment patient demographics are shown in Table 1. Patients were predominantly male (52.6%) and nonsmokers (56.8%). Median age at diagnosis was 57 years (range, 28–80). Most patients (75.9%) presented with a KPS >70. More than half of the patients had ≥2 brain lesions. Approximately 73.4% had uncontrolled primary tumors and 61.8% presented with extracranial metastases. In total, 107 patients (29.6%) were treated with radiotherapy only (77 patients with WBRT, 20 with SRS, and 10 with SRS plus WBRT), 71 (19.7%) received an EGFR-TKI or ALK-TKI only (76.1% of these had EGFR or ALK mutations), and 55 (15.2%) were treated with RT plus an EGFR-TKI (EGFR-TKI concurrently or sequentially with radiotherapy). The remaining 63 patients received chemotherapy. Sixty-five patients received steroid treatment or best supportive care.

**Table 1:** Basic demographics

Variable	Number of patients	%
Gender		
Male	190	52.6
Female	171	47.4
Age(years)		
median(range)	57 (28-80)	
KPS		
<70	87	24.1
70-80	235	65.1
90-100	39	10.8
Smoking history		
Never	205	56.8
Ever	156	43.2
KRAS		
Positive	7	1.9
Negative	107	29.6
unknown	247	68.4
ALK		
Positive	10	2.8
Negative	96	26.6
unknown	255	70.6
EGFR		
Positive	160	44.3
Negative	201	55.7
NO. of brain metastases		
1	131	36.3
2	74	20.5
3	32	8.9
4	49	13.6
≥5	75	20.8
Extracranial metastases		
Yes	223	61.8
No	138	38.2
Control of primary tumor		
Yes	96	26.6
No	265	73.4
WBRT/SRS/WBRT+SRS		
Yes	162	44.9
No	199	55.1
TKIs		
Yes	126	34.9
No	235	65.1
Chemotherapy		
Yes	63	17.5
No	298	82.5

KPS, Karnofsky performance status; KRAS, V-Ki-ras2 Kirsten rat sarcoma viral oncogene homolog; ALK, anaplastic lymphoma kinase-rearranged; EGFR, epidermal growth factor receptor; WBRT, whole brain radiotherapy; SRS, stereotactic radiosurgery; TKIs, tyrosine kinase inhibitors.

### Overall survival and prognoses

**Figure 1:**
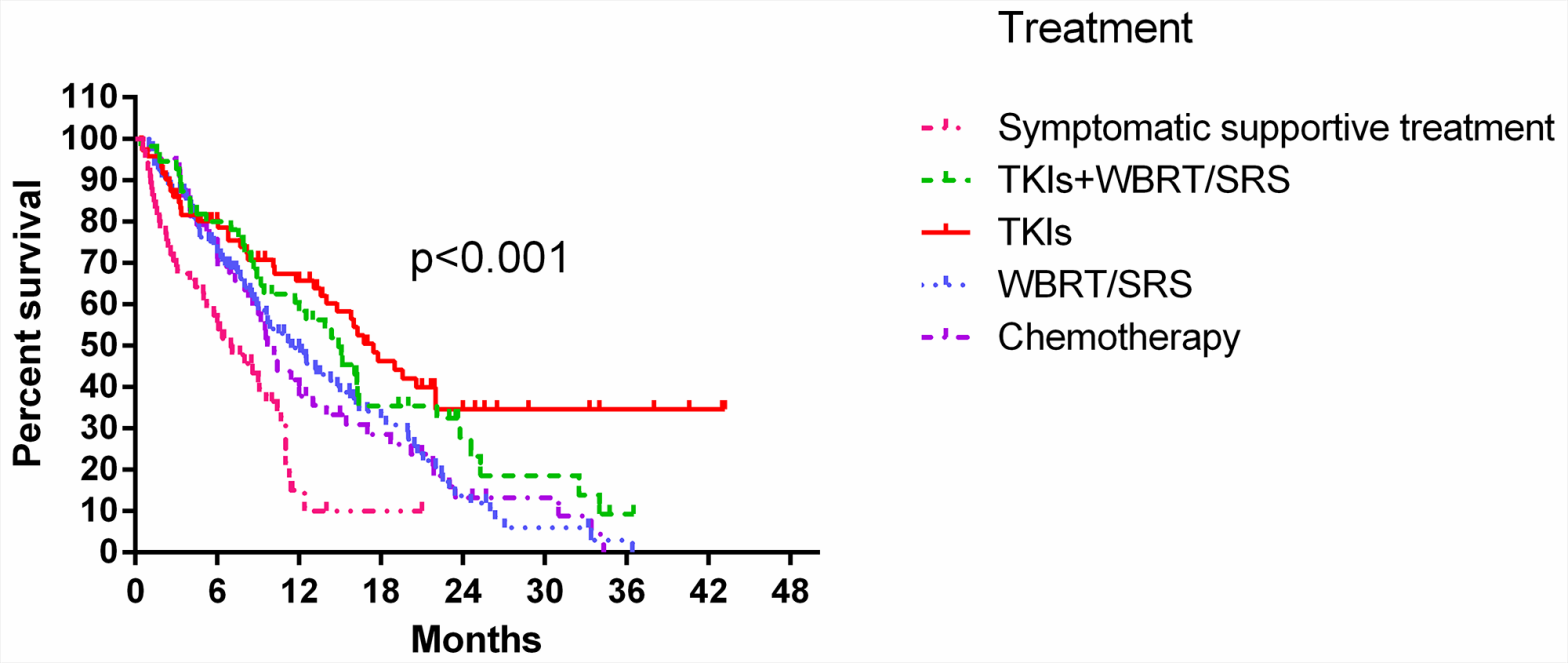
Kaplan-Meier curves showing OS by treatment modality

At the last follow-up in January 2017, 112 patients (31.0%) were still alive. Median survival time was 11.3 months (95% CI: 9.596–13.004), and one-year survival rate was 46.9%. Potential OS predictive factors are shown in Table 2. In univariate analyses, pretreatment prognostic factors predicting better OS included female gender, age <65 years, EGFR or ALK mutation, high KPS, and no extracranial metastases. However, only female gender was significant (*P*=0.055). Of the various treatments, EGFR-TKIs resulted in the best OS (Figure 1). In multivariate analyses, OS predictive factors included age, EGFR or ALK status, KPS, and targeted therapy.

**Table 2:** Results of the univariate and multivariate analysis of survival

Variables	Univariate analysis	Multivariate analysis		
	P	Risk ratio	95% CI	P
Gender	0.055			
Female vs. male				
Age	0.048	0.703	0.523 to 0.945	0.02
≥65 vs. <65				
Gene status	<0.001	0.692	0.519 to 0.922	0.012
EGFR pos or ALK pos				
EGFR neg and ALK neg/unk				
Karnofsky Performance Score	<0.001	0.065	0.040 to 0.105	<0.001
<70 vs. 70-80 vs. 90-100				
Extracranial metastases	0.017			
Yes vs. no				
TKIs	<0.001	0.718	0.527 to 0.978	0.036
Yes vs. no				

EGFR, epidermal growth factor receptor; ALK, anaplastic lymphoma kinase-rearranged; TKIs, tyrosine kinase inhibitors; CI, confidence interval.

### Prognostic indexes

**Figure 2:**
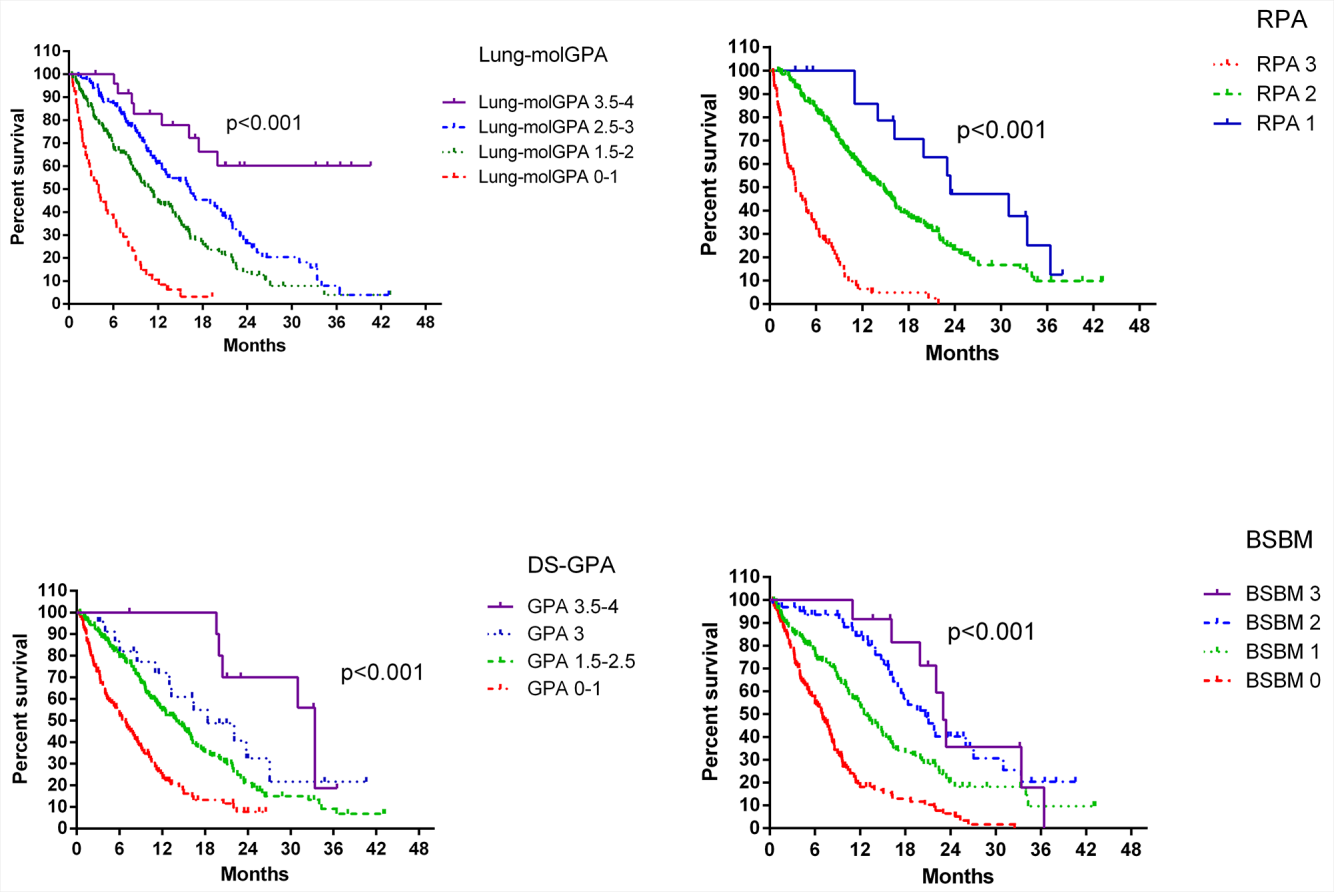
OS according to each of the four studied indexes

Prognostic values for all four indexes are shown in Figure 2 and Table 3. In the log-rank test, all four indexes predicted OS (*P*<0.001). Harrell^’^s C indexes were 0.732, 0.729, 0.724, and 0.747 for RPA, DS-GPA, BS-BM, and Lung-molGPA, respectively. All four systems appeared to offer medium predictive values, with Lung-molGPA performing best.

**Table 3:** Distribution of the study population and median overall survival according to the class of prognostic scores; Harrell’s concordance index (HCS)

	Number of pts (%)	MOS (95% CI)	P	Harard ratio (95% CI)	HCS (95% CI)
RPA					0.732 (0.691-0.772)
1	17 (4.7)	23.44M (10.889-35.991)		1	
2	257 (71.2)	14.8M (12.62-16.98)	<0.001	1.945 (1.021-3.705)	
3	87 (24.1)	3.37M (2.276-4.464)		10.185 (5.136-20.195)	
DS-GPA					0.729 (0.689-0.770)
3.5-4	11 (3.0)	33.4M (31.35-35.45)		1	
3	23 (6.4)	18.37M (8.458-28.282)	<0.001	1.776 (0.673-4.684)	
1.5-2.5	206 (57.1)	14M (11.454-16.546)		2.784 (1.221-6.348)	
0-1	121 (33.5)	6.4M (4.727-8.073)		6.230 (2.692-14.414)	
BSBM					0.724 (0.684-0.764)
3	12 (3.3)	23M (21.16-24.84)		1	
2	63 (17.5)	20.67M (16.245-25.095)		1.044 (0.480-2.274)	
1	145 (40.2)	12.53M (10.376-14.684)	<0.001	2.084 (1.009-4.306)	
0	141 (39.1)	7M (5.641-8.359)		4.887 (2.363-10.110)	
Lung-molGPA					0.747 (0.706-0.787)
3.5-4	25 (6.9)	17M		1	
2.5-3	117 (32.4)	16.3M (11.229-21.371)	<0.001	2.907 (1.396-6.055)	
1.5-2	165 (45.7)	11M (9.208-12.792)		4.629 (2.243-9.551)	
0-1	54 (15)	4M (2.441-5.559)		14.138 (6.571-30.418)	

CI, confidence interval; MOS, median overall survival; M, months; Pts, patients.

## DISCUSSION

Prognostic indexes are important tools for estimating prognosis and tailoring treatments for individual patients with BMs. Such indexes are usually established by summing the prognostic weights of independent factors in Cox regression models and then dividing these into risk zones. However, independent prognostic factors vary depending on the primary tumor site, and other biological makers may also play roles. The recently published NSCLC-specific Lung-molGPA index is the first to incorporate both traditional factors, such as patient age and gender, and EGFR and ALK mutation status [16, 17]. Our assessment showed that the Lung-molGPA index offers medium OS predictive ability in lung adenocarcinoma patients with BMs.

In our study, all four of the published prognostic indexes assessed could predict patient prognosis, demonstrating the reliability and clinical relevance of these scores. However, the Lung-molGPA is the first prognostic system, updated from the lung-DS-GPA, that includes potential biomarker statuses. Harrell^’^s concordance indexes ranged from 0.724–0.747 (medium predictive ability) for the four indexes, with the Lung-molGPA performing best.

However, multivariate analysis results in the present study did not totally agree with those of Sperduto, *et al.* [16]. Independent pretreatment prognostic factors in the Cox regression model were KPS, EGFR and ALK status, and age. In our study, number of metastases and control of primary tumor, which are included in the Lung-molGPA index, were not significant. We suggest three possible reasons for these discrepancies. First, treatments in the present study differed from those in the study by Sperduto, *et al.*, in which all BM patients were treated with WBRT or SRS. In our study, 34.9% of patients received targeted therapy, which systematically treated both cranial and extracranial lesions [18]. Therefore, number of metastases and control of primary tumor might not appear significant in our multivariate analysis. Targeted therapy is now widely recognized as the first-line treatment for patients with EGFR mutations [19-21], and radiotherapy appears to offer these patients no survival benefit [22, 23]. Our study also showed that EGFR-TKI treatment alone was the most effective of the options evaluated via log-rank test. Our multivariate analysis, which included both pretreatment and treatment factors, showed that both gene mutation status and targeted therapy could predict prognosis. This confirms the need to incorporate gene statuses into prognostic indexes for lung adenocarcinoma patients with BMs.

Second, due to EGFR mutation variations between Chinese and western patients, the present study had a higher EGFR mutation rate than the Sperduto, *et al.* study (44.3% vs. 29%) [24, 25]. Thus, more patients in our study were treated with EGFR-TKIs, potentially impacting our results. Finally, Sperduto, *et al.* reported that only 54% of patients (816/1,521) had confirmed EGFR and/or ALK alternations. This finding might have reduced the weight of gene alterations and thus affected Cox regression analysis results. Our findings suggest that identification of novel independent pretreatment prognostic factors would increase NSCLC BM patient prognostic index accuracies.

Our study had several limitations. Initially, only 106 patients had been tested with ALK mutations status. This may underscore the effects of this gene alteration in multivariate analysis. Additionally, the number of non-adenocarcinoma NSCLC patients with gene mutations was small, and this subgroup was not studied. The EGFR mutation rate in patients without adenocarcinoma of the lung is <5% [26]. In our study, 11/47 non-adenocarcinoma patients with BMs (23.4%) had EGFR mutations. A specific prognostic system for this patient subgroup is needed.

In conclusion, we confirmed that the Lung-molGPA index could be useful in estimating OS in patients with BMs from lung adenocarcinoma. The Lung-molGPA appeared to offer the most accurate predictions of the four systems assessed. However, the independent prognostic factors found in our study did not entirely match those of the Lung-molGPA index. In the era of targeted therapy, this index must be further updated to incorporate more specific prognostic factors based on additional patient data.

## MATERIALS AND METHODS

### Patient eligibility

A total of 2,823 patients with NSCLC and EGFR mutations were identified between January 2010 and August 2016 from our lung cancer medical database. All EGFR mutations were identified by direct DNA sequencing or amplification refractory mutation system (ARMS). Some patients were also tested for ALK mutations by polymerase chain reaction (PCR) or immunohistochemical (IHC) staining. Patients selected for this study had been diagnosed with lung adenocarcinoma and BMs via computed tomography (CT) and/or magnetic resonance imaging (MRI) at diagnosis or during the course of treatment. Patients with synchronous lung cancer and other cancers were excluded. Patient clinical and treatment data, follow-up examination results, and/or date of death were available. As a result, 408 patients were diagnosed with BMs. Thirty-one patients with squamous cell lung cancer, six with neuroendocrine carcinoma, four with small cell lung cancer, two with synchronous lung cancer and esophageal carcinoma, one with breast cancer, and one with cervical cancer were excluded from the study. Two additional patients were excluded due to incomplete clinical data. In total, 361 patients met the selection criteria. Patient-related variables included age, gender, KPS, primary lesion status (controlled vs. uncontrolled), extracranial systemic metastases (present or absent), number of BMs (single vs. multiple), gene mutation status (EGFR- or ALK-positive vs. EGFR-negative and ALK-negative/unknown). Treatment options included WBRT, stereotactic radiosurgery (SRS), management with EGFR-TKIs (tyrosine kinase inhibitors; gefitinib or erlotinib), chemotherapy, and targeted therapy and radiotherapy combined. The Institutional Review Board of Shanxi Cancer Hospital approved the study.

The primary endpoint of this study was overall survival (OS), defined from the date of BM diagnosis to death or last follow-up. OS and prognostic factors were evaluated in uni- and multivariate analyses. Patients were stratified according to RPA, DS-GPA, and BS-BM criteria separately. RPA, GPA, BS-BM, and NSCLC-specific GPA parameters are shown in Table 4. Differences between the systems and their predictive values were evaluated.

**Table 4:** Clinical parameters used for 4 prognostic indexs (RPA, DS-GPA, BSBM, and Lung-molGPA)

	RPA		
Class 1	Age<65 y,KPS≥70, controlled primary tumor, no extracranial metastases		
Class 2	All patients not in Class 1 or 3		
Class 3	KPS<70		
	**DS-GPA**		
	0	0.5	1
Age,y	>60	50-59	<50
KPS	<70	70-80	90-100
Number of BM	>3	2-3	1
ECM	Yes		No
	**BSBM**		
	0		1
KPS	50-70		80-100
Control of primary tumor	No		Yes
ECM	Yes		No
	**Lung-molGPA**		
	0	0.5	1
Age,y	≥70	<70	
KPS	<70	70-80	90-100
ECM	Yes		No
Number of BM	>4	1-4	
Gene status	EGFR neg/unk and ALK neg/unk		EGFR pos or ALK pos

RPA, recursive partitioning analysis; DS-GPA, diagnosis-specific graded partitioning analysis; BSBM, basic score for brain metastases; BM, brain metastases; KPS, Karnofsky performance status.

### Statistical analysis

OS was estimated from the first day of BM diagnosis to death or date of last follow-up using the Kaplan-Meier method. Subgroup analysis was performed using the log-rank test for univariate analyses and the Cox proportional hazards model for multivariate analyses. *P*<0.05 was considered significant. Harrell^’^s C index was used to estimate the discriminative abilities of the four indexes. All analyses were performed using SPSS statistical software 17.0 and R v. 2.15.2 [27].
